# Physician Wellness During a Pandemic

**DOI:** 10.5811/westjem.2020.7.48472

**Published:** 2020-09-24

**Authors:** Kevin Fitzpatrick, Rachel Patterson, Krista Morley, Jill Stoltzfus, Holly Stankewicz

**Affiliations:** St. Luke’s University Health Network, Department of Emergency Medicine, Bethlehem, Pennsylvania

## Abstract

**Introduction:**

We are currently in the midst of the coronavirus disease 2019 (COVID-19) pandemic. Research into previous infectious disease outbreaks has shown that healthcare workers are at increased risk for burnout during these dire times, with those on the front lines at greatest risk. The purpose of this prospective study was to determine the effect that the COVID-19 pandemic has had on the wellness of emergency physicians (EP).

**Methods:**

A survey was sent to 137 EPs in a multi-hospital network in eastern Pennsylvania. We compared 10 primary and two supplemental questions based on how the physicians had been feeling in the prior 2–3 weeks (COVID-19 period) to the same questions based on how they were feeling in the prior 4–6 months (pre-COVID-19 period).

**Results:**

We received 55 responses to the survey (40.1% response rate). The study found that during the pandemic, EPs felt less in control (p-value = 0.001); felt decreased happiness while at work (p-value 0.001); had more trouble falling asleep (p-value = 0.001); had an increased sense of dread when thinking of work needing to be done (p-value = 0.04); felt more stress on days not at work (p-value <0.0001); and were more concerned about their own health (p-value <0.0001) and the health of their families and loved ones (p-value <0.0001).

**Conclusion:**

This study showed a statistically significant decrease in EP wellness during the COVID-19 pandemic when compared to the pre-pandemic period. We need to be aware of evidence-based recommendations to help mitigate the risks and prevent physician burnout.

## INTRODUCTION

Severe acute respiratory syndrome coronavirus 2 (SARS-CoV-2), a novel coronavirus that appeared in Wuhan, China, in late 2019, was named a public health emergency of international concern by the World Health Organization (WHO) on January 30, 2020.^1^ Less than two months later, on March 11, 2020, the WHO declared it a global pandemic.^1^ SARS-CoV-2 and the disease it causes, coronavirus disease 2019 (COVID-19), have now infected millions worldwide, and the related death toll currently numbers in the hundreds of thousands.^2^ The pandemic continues to infect tens of thousands daily as physicians in emergency departments (ED) relentlessly battle the virus on the front lines.^2^

From the very initial stages of the outbreak, the United States’ response has been rightfully focused on the availability of personal protective equipment (PPE), ventilators, and other medical supplies as evidenced by the US government invoking the Defense Production Act.^3^,^4^ However, this battle, as outbreaks from the past have shown, comes at a great cost to physician wellness, and if not given the attention it appropriately deserves, can subsequently lead to burnout.^5^–^7^ Physician wellness includes physical, mental, and social well-being balanced between personal and work-life domains.^8^ It has been well established that physician wellness and burnout have a direct impact on patients in terms of quality of care and patient safety as well as on the medical providers themselves.^9^ Burnout is more common in physicians than with other US workers, and emergency physicians (EP) are among those at greatest risk.^10^

Research into previous outbreaks of influenza, H1N1, SARS, and MERS, has shown that burnout is commonly experienced by healthcare workers. 5–7 Multiple factors are at play including fear and anxiety over an unknown number of infected, excessive workload, lack of resources, insomnia, and isolation.^5^–^7^ The effect of working in this constantly changing environment has been shown to be particularly stressful, and those working in high-risk units experienced greater levels of distress.^7^ Lin et al found that ED staff faced more demanding work conditions as well as more physical and psychological stress than staff in other units.^6^

The purpose of this prospective study was to determine the effect that the COVID-19 pandemic has had on the wellness of EPs. Our primary objective was to compare physician wellness during the pandemic to physician wellness pre-pandemic. Our secondary objective was to compare the time spent using social media and consuming news during the pandemic to the time spent pre-pandemic.

## METHODS

This was a prospective survey study administered to EPs in an 11-hospital network located in eastern Pennsylvania, about 80 miles from New York City. The survey was sent to 137 physicians via a secure hospital email. Fifty-five physicians responded via e-mail to the research assistant who then assigned participant numbers to each physician to provide anonymity. The survey (Figure) was partially derived from previously validated surveys.^11^ The survey asked 10 primary questions and two supplemental questions regarding physician wellness, and participants were asked to answer questions based on how they have been feeling over the prior 2–3 weeks (March 27–April 17, 2020), which correlated to the beginning of the Covid-19 pandemic in our area.

The subjects were asked to answer questions using a scale for the primary questions ranging from not at all (1) to completely true (5), and for the supplemental questions ranging from 0 to 1 hours (1) to greater than 5 hours (4). To serve as a baseline for comparison, the physicians were then asked to answer the same primary and supplemental questions based on how they thought they felt 4–6 months before the start of the pandemic. Due to the skewed and ordinal nature of our survey questions, we conducted separate Wilcoxon signed-rank tests. We analyzed our data using SPSS version 25 (IBM Corporation, Armonk, NY) and reported medians and ranges for all survey outcomes, with p <.05 denoting statistical significance and no adjustment for the multiple comparisons.

## RESULTS

A total of 55 subjects (40.15% response rate), 39 male and 16 female, completed the survey. Of the 39 male subjects, 17 were resident physicians and 22 were attending physicians. The 16 female subjects included six resident physicians and 10 attending physicians. We collected age data in ranges by decade with a median age range of 30–40 years.

Wilcoxon signed-rank test analysis showed a statistically significant difference between the five-point scale score distributions of the pre-COVID-19 period and the COVID-19 period in seven out of the 10 primary questions. There was no statistically significant difference in three out of the 10 primary questions (Table) Likewise, there was no statistically significant difference in either of the two supplemental questions (Table).

The data showed that during the pandemic, EPs felt less in control (p-value = 0.001) and felt decreased happiness while at work (p-value = 0.001). Additionally, during the pandemic, they had more trouble falling asleep (p-value = 0.001) and had an increased sense of dread when thinking of work needing to be done (p-value = 0.04). Furthermore, the data revealed that during the pandemic, EPs felt more stress on days not at work (p-value <0.0001) and were more concerned about their own health (p-value <0.0001) as well as the health of their families and loved ones (p-value <0.0001).

## DISCUSSION

The COVID-19 pandemic has forced many healthcare workers to confront challenges that they have never experienced before. This unprecedented time is fraught with fear and anxiety especially for frontline workers providing direct patient care. A crucial yet often overlooked aspect of the public health response to the pandemic is physician wellness. This prospective survey study conducted at an early stage in the COVID-19 pandemic provides important insight into this marginalized aspect of the global response.

Our study revealed that there was an overall decrease in EP wellness during the COVID-19 pandemic when compared to the pre-pandemic period. The data showed a statistically significant difference in seven out of the 10 primary wellness survey questions. The difference indicated a decrease in wellness during the pandemic for all seven of the questions that showed statistical significance. These findings are in line with findings regarding physician wellness from previous infectious disease outbreaks.^5^–^7^ Research into past outbreaks also showed that physician concern for their own health (p-value <0.0001) and concern for family and loved ones (p-value <0.0001) was common, which was echoed in this study.^5^–^7^

Additionally, the study showed that there was no difference during the pandemic compared to the pre-pandemic period in physicians staying asleep, concern about their financial situation, and, interestingly, feelings of stress at work. However, feeling stress on days not at work did significantly increase during the pandemic (p-value <0.0001). This difference is likely multifactorial but may partially be explained by inadequate social support due to increased isolation as well as mandated school closures affecting work-life balance. Another intriguing finding of our study was that, despite the constant media coverage, subjects did not significantly increase the amount of time spent viewing news or using social media. The decreased physician wellness scores during the pandemic were therefore independent of these activities.

There is a need for larger studies on physician wellness during the COVID-19 pandemic, but the findings of this study could inform medical administration about the need for protective measures, not only in the form of masks and gowns but also in the form of developing programs to address physician wellness and burnout. Initial evidence-based recommendations are emerging to address these concerns at the organizational, team, and individual levels.^12^–^16^ If we do not take these recommendations seriously and implement the needed safeguards, we could soon be dealing with another outbreak – physician burnout.

## LIMITATIONS

This study has several limitations. The sample size (n = 55) was relatively small. Our study group originated from a single hospital network, was a convenience sample, and was limited by non-response bias. Survey questions were derived from a previously validated study, but the specific question that subjects answered might not have covered the broad range of physician wellness. The survey used physician self-report of feelings up to six months earlier, which introduced the potential for recall bias, as well as social-desirability bias. Even though statistical significance was found in several of the questions, there may not be a clinical significance given how similar the medians and/or general distribution of scores were in some cases. Future studies will attempt to conduct multivariable modeling to tease out independent predictors of survey responses, such as gender or level of training of the physician, provided sample size is sufficient.

## CONCLUSION

In keeping with data from past outbreaks, this prospective survey study showed that there was an overall decrease in emergency physician wellness during the COVID-19 pandemic when compared to the pre-pandemic period. Evidence-based recommendations to address this often- overlooked issue are starting to emerge, and it is crucial that individual physicians, as well as hospital administrators, be aware of these safeguards in order to prevent unnecessary physician burnout.

## Figures and Tables

**Figure:**
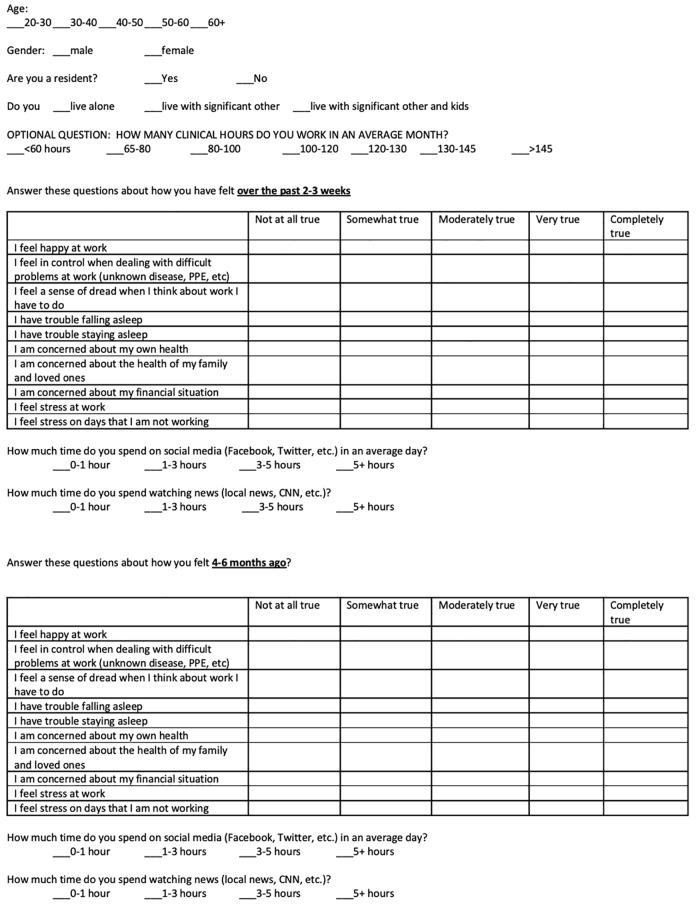
Wellness survey of emergency physicians during the COVID-19 pandemic.

**Table:** Statistical analysis of primary and supplemental survey questions.

Primary Survey Questions (n = 55)	Pre-COVID-19 (median, range)	COVID-19 (median, range)	P-value
I feel happy at work.	4 (1–5)	3 (1–5)	0.001
I feel in control when dealing with difficult problems at work (unknown disease, PPE, etc.).	4 (1–5)	3 (1–5)	0.001
I feel a sense of dread when I think about work I have to do.	1 (1–4)	2 (1–5)	0.04
I have trouble falling asleep.	1 (1–5)	2 (1–5)	0.001
I have trouble staying asleep	1 (1–5)	1 (1–5)	N/A
I am concerned about my own health.	1 (1–5)	2 (1–5)	<.0001
I am concerned about the health of my family and loved ones.	2 (1–5)	4 (1–5)	<.0001
I am concerned about my financial situation.	2 (1–5)	2 (1–5)	N/A
I feel stress at work	2 (1–5)	2 (1–5)	N/A
I feel stress on days that I am not working.	1 (1–4)	2 (1–5)	<.0001

Supplemental Survey Questions (n = 55)			

Social media (hours/day)	1 (1–3)	1 (1–3)	N/A
Watching news (hours/day)	1 (1–3)	1 (1–2)	0.06

*COVID-19*, coronavirus 2019; *PPE*, personal protective equipment.

## References

[1] World Health Organization (2020). Coronavirus Disease (COVID-19) - events as they happen.

[2] World Health Organization WHO Coronavirus Disease (COVID-19) Dashboard.

[3] The White House https://www.whitehouse.gov/presidential-actions/memorandum-order-defense-production-act-regarding-purchase-ventilators/.

[4] The White House Memorandum on Order Under the Defense Production Act Regarding 3M Company.

[5] Maunder R, Hunter J, Vincent L (2003). The immediate psychological and occupational impact of the 2003 SARS outbreak in a teaching hospital. CMAJ.

[6] Lin C-Y, Peng Y-C, Wu Y-H (2007). The psychological effect of severe acute respiratory syndrome on emergency department staff. Emerg Med J.

[7] Styra R, Hawryluck L, Robinson S (2008). Impact on health care workers employed in high-risk areas during the Toronto SARS outbreak. J Psychosom Res.

[8] Brady KJS, Trockel MT, Khan CT (2018). What do we mean by physician wellness? A systematic review of Its definition and measurement. Acad Psychiatry.

[9] Bodenheimer T, Sinsky C (2014). From triple to quadruple aim: care of the patient requires care of the provider. Ann Fam Med.

[10] Shanafelt TD, Boone S, Tan L (2012). Burnout and satisfaction with work-life balance among US physicians relative to the general US population. Arch Intern Med.

[11] Trockel M, Bohman B, Lesure E (2018). A brief instrument to assess both burnout and professional fulfillment in physicians: reliability and validity, including correlation with self-reported medical errors, in a sample of resident and practicing physicians. Acad Psychiatry J.

[12] Walton M, Murray E, Christian MD (2020). Mental health care for medical staff and affiliated healthcare workers during the COVID-19 pandemic. Eur Heart J Acute Cardiovasc Care.

[13] Shah K, Chaudhari G, Kamrai D (2020). How essential is to focus on physician’s health and burnout in coronavirus (Covid-19) pandemic?. Cureus.

[14] Shah K, Kamrai D, Mekala H (2020). Focus on mental health during the coronavirus (COVID-19) pandemic: applying learnings from the past outbreaks. Cureus.

[15] Sasangohar F, Jones SL, Masud FN, Vahidy FS, Kash BA (2020). Provider burnout and fatigue during the COVID-19 pandemic: lessons learned from a high-volume intensive care unit. Anesth Analg.

[16] Sultana A, Sharma R, Hossain MM (2020). Burnout among healthcare providers during COVID-19 pandemic: challenges and evidence-based interventions. IJME.

